# The First Eighteen Months of African Swine Fever in Wild Boar in Saxony, Germany and Latvia—A Comparison

**DOI:** 10.3390/pathogens12010087

**Published:** 2023-01-05

**Authors:** Michael Richter, Katja Schulz, Tobias Elflein, Jens Achterberg, Edvīns Oļševskis, Mārtiņš Seržants, Kristīne Lamberga, Franz Josef Conraths, Carola Sauter-Louis

**Affiliations:** 1 Department 25, Veterinary Affairs and Food Control, Landesdirektion Sachsen, 01099 Dresden, Germany; 2 Institute of Epidemiology, Friedrich-Loeffler-Institut, Federal Research Institute for Animal Health, 17493 Greifswald-Insel Riems, Germany; 3 Saxon State Ministry for Social Affairs and Cohesion, 01097 Dresden, Germany; 4 Food and Veterinary Service, LV-1050 Riga, Latvia; 5 Institute of Food Safety, Animal Health and Environment “BIOR”, LV-1076 Riga, Latvia

**Keywords:** epidemiology, prevalence, wild boar population density, spread, control measures

## Abstract

African swine fever (ASF) emerged in Latvia in 2014. In 2020, the virus has been detected in the German federal state, Saxony. In both regions, the virus was probably introduced by infected wild boar coming from affected neighboring countries. As the current ASF control strategy at EU level had not yet been developed at the time of ASF introduction into Latvia, disease control measures in both study areas differed over time. Assessing the course of ASF in Saxony and the implemented control strategies, the first 18 months of the ASF epidemic were epidemiologically compared between Saxony and Latvia. ASF wild boar surveillance data were analyzed and the prevalence of ASF virus-positive wild boar was estimated. For estimating the wild boar density, the numbers of wild boar per km² were calculated for the respective geographical areas. The number of samples collected from hunted wild boar and wild boar found dead was higher in Saxony. The ASF virus prevalence in Latvia was significantly higher than in Saxony, indicating that Saxony has had more time for getting prepared for dealing with an ASF incursion. Experience from other countries and the rapid implementation of new control strategies may have helped Saxony deal with ASF.

## 1. Introduction

African swine fever (ASF) has, in recent years, become a threat to the global pig industry. The disease is caused by a large DNA virus that has no zoonotic potential and affects only members of the Suidae family [1]. In Eastern and Central Europe, infected wild boar populations play a major role in the epidemiology of the disease and constitute an important risk factor for disease introduction into domestic pig holdings [2,3,4,5]. Within the European Union (EU), the occurrence of ASF was restricted to Sardinia for many years. This changed after the disease entered the Baltic states and Poland in 2014 [6]. In Latvia, ASF of genotype II emerged in the wild boar population in June 2014, probably introduced by immigration of infected wild boar from an affected neighboring non-EU country, presumably Belarus [4]. Although ASF of this genotype had already been introduced into Georgia in 2007 and constituted thus a more tangible threat to countries in the EU, the disease emergence in 2014 hit Latvia relatively unexpectedly. In Belarus, only in 2013 but not in 2014, ASF-positive wild boar or outbreaks in domestic pig holdings were reported. Still, the first infected wild boar in Latvia were detected on the border with this country. Several control measures were swiftly put in place in Latvia. Among others, incentives were paid for hunting wild boar with the aim of reducing the ASF-susceptible population. The collection and safe disposal of detected wild boar carcasses was initiated to remove infection sources from the environment [7].

Germany started to increase ASF surveillance activities and to prepare control measures since 2014. At the latest, the introduction of ASF into west Poland in November 2019 [8] put the German veterinary authorities on alert. Thus, the detection of an ASF-positive wild boar carcass in the German federal state of Brandenburg in September 2020 was not completely unexpected [9]. The size of the affected area in western Poland, and the constant infection pressure across the long common border between Poland and Germany caused several independent ASF clusters in wild boar in eastern Germany within a couple of weeks or months [10]. Although an electric fence built close to the border between Poland and Saxony on German territory may have delayed the ASF entry at least to some extent, it was not surprising that the first ASF cases appeared in Saxony by the end of October 2020. After the first detection of an ASF-positive wild boar in the district of Görlitz, eastern Saxony, intensive carcass searches were organized. In addition, further fences were built to reduce or delay further disease spread. One year after the first detection of ASF in Saxony, the disease emerged in the district of Meißen, 65 km westwards to the initially affected region in the East. Due to the large distance and the usually expected speed of ASF spread (between the median of 8.2 and 16.3 km/year) [11], it was assumed that the cases in the district of Meißen were due to a separate introduction of ASF into this region, possibly by human activity [12].

Due to the apparently similar first introduction of ASF into both study areas through wild boar migration but also due to constant infection pressure from neighboring countries (in particular Belarus in the case of Latvia and Poland in the case of Saxony), we aimed to compare the first 18 months of the ASF epidemic in Saxony and Latvia. Although the emergence of ASF in Latvia almost seven years before the one in Germany was due to a similar introduction pathway, the conditions in 2014 were different. Thus, we also aimed to evaluate and discuss the effect of the implementation of control measures, which had been developed over time, also in the Czech Republic and in Belgium, the only countries that managed to eliminate ASF from wild boar in affected regions so far [13,14]. Thus, the study may help to assess the epidemiological situation, surveillance and control measures in a newly affected region such as Saxony and to revise the currently implemented measures if necessary.

## 2. Materials and Methods

### 2.1. Surveillance Data

For the analyses, ASF wild boar surveillance data from the German federal state of Saxony and from Latvia were used. Data from the first 18 months of the epidemic were analyzed. Consequently, Saxon data were used from 1 November 2020 through to 27 April 2022 and Latvian data were used from 25 June 2014 through to 30 November 2015.

Surveillance data were retrieved from the CSF/ASF wild boar surveillance database of the European Union (https://surv-wildboar.eu) (accessed on 11 July 2022). The data were used with the approval of the respective veterinary authorities in both countries. Each record referred to a single wild boar and included information about the age of the sampled animal, the test result (serology and/or virology), the date of sampling and the origin of the sample (from apparently healthy, hunted wild boar (active surveillance) or from wild boar shot sick, found dead or died through a road traffic accident (RTA) (passive surveillance)).

All data were analyzed descriptively. Data records originating from wild boar found dead, shot sick and killed in an RTA were merged and analyzed together. Thus, in the present study, all these samples were summarized under wild boar found dead/passive surveillance. In Saxony, 617 data records had no information on the origin of the sample, these samples were assigned to the samples originating from passive surveillance.

The number of investigated samples and prevalence estimates for wild boar that had tested positive for ASF virus (ASFV) were determined for both countries on the municipality level and on a monthly basis. Prevalence estimates were calculated separately for hunted wild boar and wild boar found dead. The estimates were generated by dividing the number of wild boar (either hunted or found dead) that had tested positive for ASFV by PCR by the total number of sampled wild boar (either hunted or found dead). The differences in the ASFV prevalence estimates in hunted wild boar and wild boar found dead between Saxony and Latvia was statistically compared using the Mann–Whitney U-Test test. A *p*-value < 0.05 was considered statistically significant.

The size of the ASF-affected municipalities was calculated by using ArcGIS ArcMap 10.8.1 (ESRI, Redlands, CA, USA). To determine the size of the affected area in the first 18 months of the epidemic, the areas of the municipalities with an ASFV prevalence above 0 were merged.

The 95% confidence intervals for the ASFV prevalence estimates per geographical area and per month were calculated according to Clopper and Pearson [15]. All analyses and figures for results on monthly basis were performed using R (https://www.r-project.org/) (accessed on 7 November 2022). Results on a geographical level were plotted on maps using ArcGIS ArcMap 10.8.1 (ESRI, Redlands, CA, USA).

### 2.2. Wild Boar Population Data

Hunting bag data from Saxony was provided on a district level and for the hunting season 2020/21. The data originated from the Saxon Game Monitoring, State Enterprise Saxony Forest, Upper Hunting Authority.

Latvian wild boar population data were estimated based on hunting bag data, which were available for each hunting management unit. For analyses, only data from the hunting season 2014/15 were used. A detailed description of this data set has been published [7]. For the comparison of the estimated wild boar population density between Latvia and Saxony, the number of wild boar/km² was used and mapped using ArcGIS ArcMap 10.8.1 (ESRI, Redlands, CA, USA).

## 3. Results

From Saxony, 38,078 data entries and from Latvia, 16,271 data records were available for analysis (Table 1). The composition of the data is presented in detail in Table 1.

**Figure 1:**
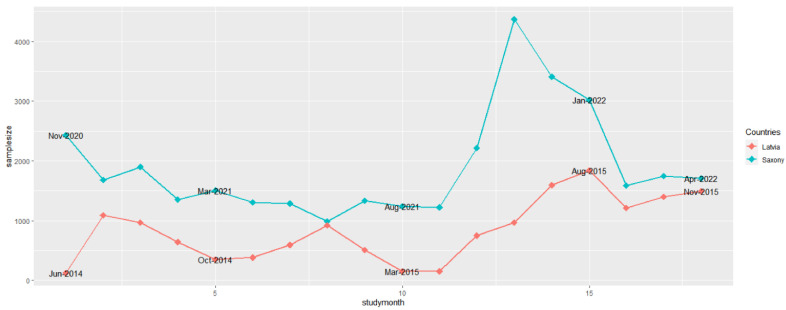
Numbers of samples from hunted wild boar (active surveillance) for African swine fever from Saxony and Latvia in the 18 study months.

In Saxony, most samples from active surveillance (4367) were examined in November 2021, 13 months after the introduction of ASF. In Latvia, tested samples from hunted wild boar peaked with 1843 specimens in August 2015, 15 months after ASFV introduction into the country (Figure 1). The lowest number of samples originating from active surveillance was taken in Latvia two months later than in Saxony. In Saxony, 986 samples were obtained in June 2021, and in Latvia, 150 samples were collected in March and April 2015. Unsurprisingly, in Latvia, the number of samples was lowest in June 2014, since the disease was first confirmed only on the 26th day of this month (Figure 1).

**Figure 2:**
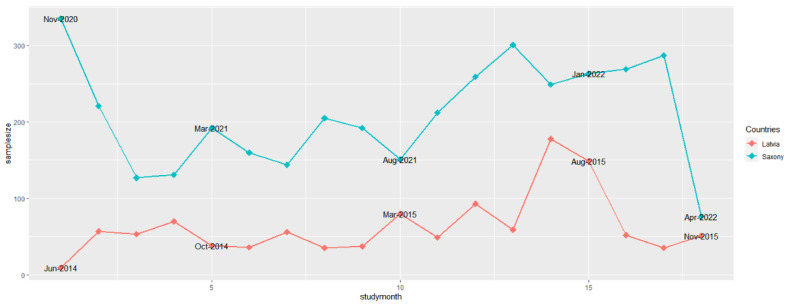
Numbers of samples from wild boar found dead (passive surveillance) for African swine fever from Saxony and Latvia in the 18 study months.

In Saxony, 617 data records had no information on the origin of the sample, these samples were assigned to the samples originating from passive surveillance. Similar to the number of samples from active surveillance, the number of samples for passive surveillance in Saxony was almost the highest in November 2021 (301). Only in the first study month (November 2020), the number of samples was slightly higher (335) (Figure 2). Similarly, the trend in the sample size obtained from passive surveillance was comparable to the sample size from active surveillance in Latvia: With 178 and 149 samples, the largest numbers of samples from passive surveillance were investigated in July and August 2015 in this country (Figure 2). In Saxony, the lowest number of samples from passive surveillance was investigated in the last month of the study period (April 2022, 76 samples). Apart from June 2014, only 35 samples were taken in Latvia in January and October 2015. However, in October and November 2014 and in February 2015, the numbers did not exceed 40 samples (Figure 2).

**Figure 3:**
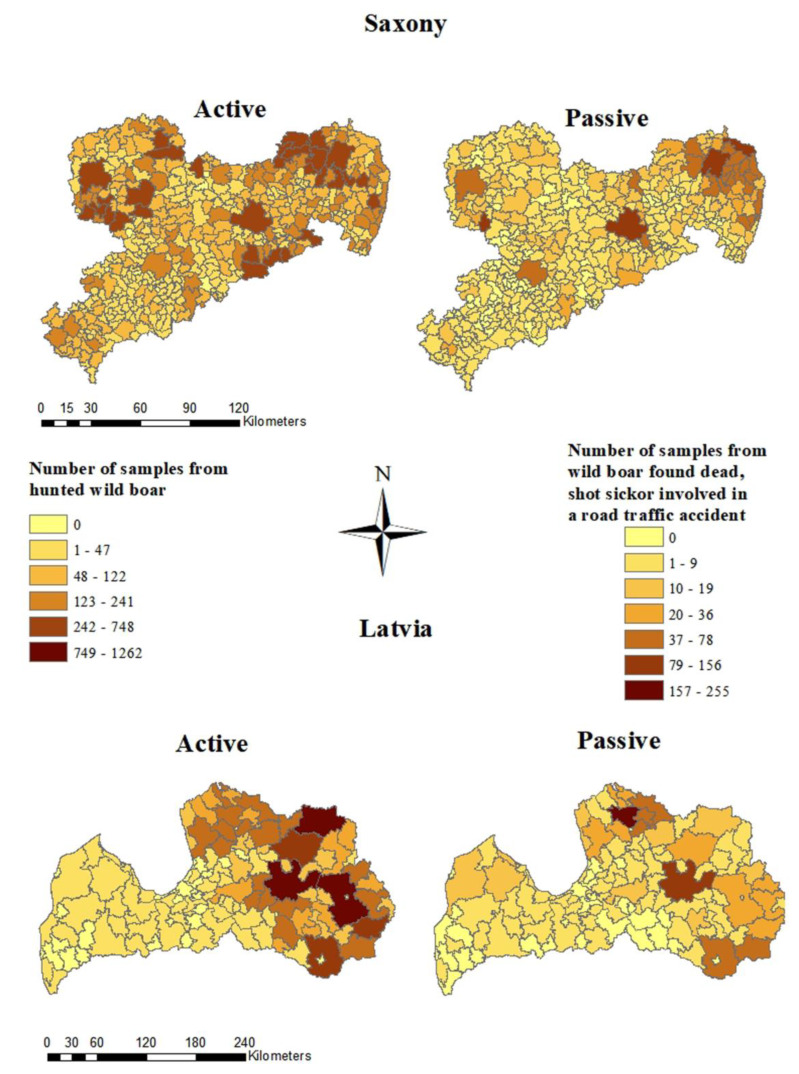
Numbers of samples from hunted wild boar (active surveillance) and wild boar found dead (passive surveillance) from Saxony and Latvia on municipality level.

In Saxony, the majority of samples from hunted wild boar and from wild boar found dead were taken in the Northeast. The largest number of samples from hunted wild boar (748 samples) and from wild boar found dead (156) were taken in the municipality of Boxberg/Oberlausitz located in the district of Görlitz. In Lohsa, located in the district of Bautzen, a large number of samples from hunted wild boar (622 samples) was investigated (Figure 3). In Borna, a town in the district of Leipzig, many (138) samples were investigated from wild boar found dead. In 195 of the 419 Saxon municipalities, less than 50 samples were taken from hunted wild boar and in 407 municipalities less than 50 samples were obtained from wild boar found dead. In 77 municipalities, no samples from wild boar found dead were investigated in the first 18 months of the ASF epidemic in Saxony (Figure 3).

In Latvia, the majority of samples from hunted wild boar were taken in the east of the country within the study period. In Rēzeknes novads and in Madonas novads, 1262 samples were collected. In 72 of 119 municipalities, less than 50 samples were investigated from hunted wild boar. Samples of wild boar found dead originated mainly from Madonas novads (121 samples) and Burtnieku novads (255 samples). In 116 of the municipalities, less than 50 samples were obtained and in 33 municipalities no samples were obtained (Figure 3).

**Figure 4:**
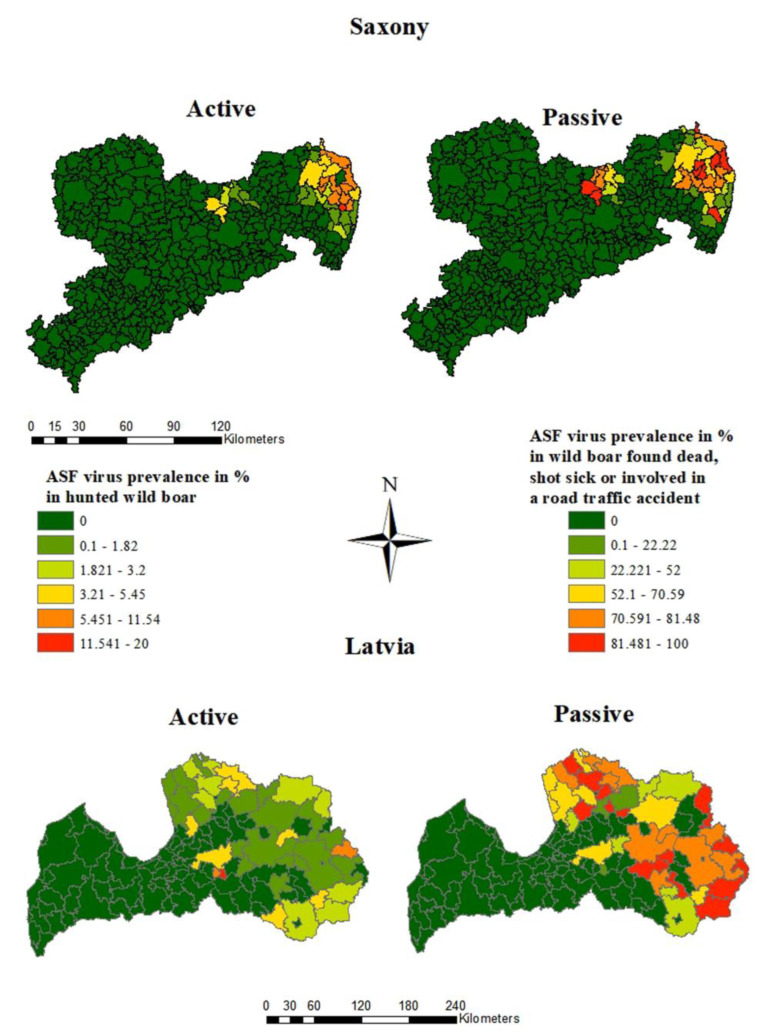
ASFV prevalence estimates in the municipalities of Saxony and Latvia in the first 18 study months of the ASF epidemic, calculated for hunted wild boar and wild boar found dead.

**Figure 5:**
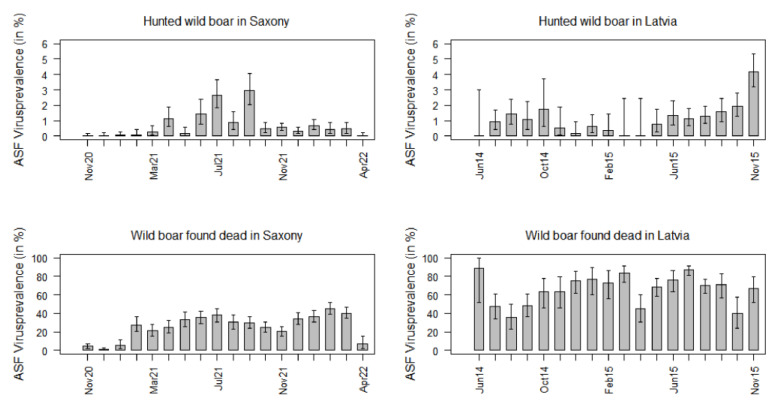
ASFV prevalence estimates in hunted wild boar and wild boar found dead in Saxony and in Latvia for each of the 18 months of the study period.

The size of the area, where ASFV-positive wild boar were detected encompassed 12% of the whole study area (2163 km^2^) in Saxony and 43% (27,538 km^2^) in Latvia. In Saxony as well as in Latvia, the ASFV prevalence estimates were clearly higher in wild boar found dead than in hunted wild boar (Figure 4 and Figure 5).

The highest prevalence estimates in hunted wild boar were found in the district of Görlitz (Figure 4), whereby the highest prevalence (19.2%; 95% CI: 10.9–30.1%) was detected in the municipality of Königshain, while the prevalence was only 4.0% (95% CI: 2.7–5.7%) in Boxberg/Oberlausitz. The highest ASFV prevalence in wild boar found dead (100.0%; 95% CI: 71.5–100.0%) was found in Ebersbach, which is located in the more centrally located district Meißen. However, 28 of the 41 affected municipalities were located in Görlitz, the district bordering Poland (Figure 4).

In Latvia, the highest ASFV prevalence in hunted wild boar (20.0%; 95% CI: 6.8–40.7% and 6.7%; 95 CI: 0.8–22.1%) was found in two small municipalities in the center of the country (Figure 4). In Ciblas novads, in the east of Latvia, the ASFV prevalence in hunted wild boar was 5.5% but with a narrower confidence interval (95% CI: 2.5–10.1%). In seven municipalities, located mainly in the east of the country, the ASFV prevalence in wild boar found dead was 100.0%. However, all 95% confidence intervals were very wide and covered at least 45.0%. In Beverīnas novads, the ASFV prevalence in wild boar found dead was 95.2% with a narrow confidence interval (83.8–99.4%). Overall, the prevalence exceeded 50.0% in 31 municipalities, with the numbers of taken samples varying greatly between municipalities (Figure 4).

In Saxony, the ASFV prevalence in hunted wild boar increased in the first few months of the study period, but it decreased significantly from September 2021 to October 2021. In the subsequent months, it did not exceed 1.0% anymore (Figure 5).

Except for a clear increase after the first three study months in February 2020, the ASFV prevalence in wild boar found dead did not change significantly over time. Yet, in April 2022, the prevalence dropped again to 7.0% (95% CI: 2.3–15.5%) (Figure 5).

Although the ASFV prevalence in Latvian hunted wild boar seemed to increase over time, only in the last study month (November 2015), the prevalence was clearly higher than in the other months (4.2%; 95% CI: 3.2–5.3%). Moreover, the ASFV prevalence in Latvian wild boar found dead did not vary considerably between the different study months (Figure 5).

The ASFV prevalence estimates in hunted wild boar did not differ significantly (*p* = 0.145) between Saxony (median 0.5%) and Latvia (median 1.0%) (Supplemental material Figure S1).

However, the prevalence estimates in Latvia (median 69.3%) were significantly higher (*p* < 0.001) than in the Saxon (median 28.8%) wild boar found dead (Supplemental material Figure S2).

**Figure 6:**
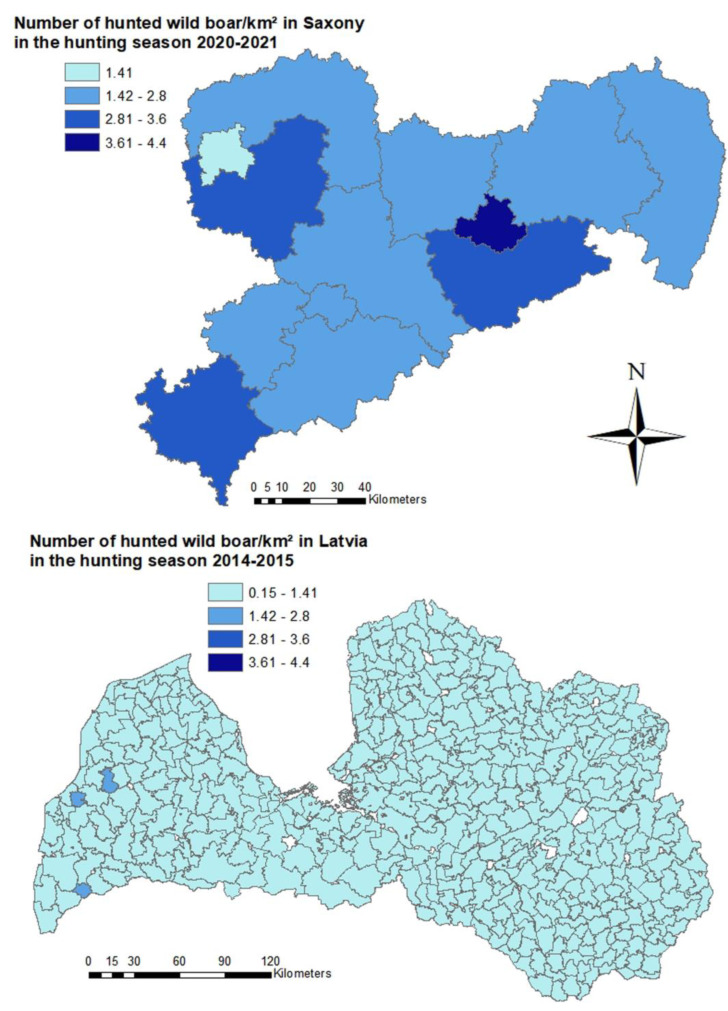
On top, estimated number of wild boar/km^2^ per district in Saxony calculated based on hunting bag data from the hunting season 2020–2021. Below, estimated numbers of wild boar/km^2^ per Latvian hunting management unit calculated on the basis of hunting bag data from the hunting season 2014–2015.

In Saxony, hunting bag data was analyzed at a district level. The number of wild boar/km^2^ was highest in the city of Dresden (4.4 wild boar/km^2^). In the Eastern districts of Görlitz and Bautzen, the density was only 2.8 and 2.5 wild boar/km^2^, respectively (Figure 6). In Latvia, the wild boar density based on hunting bag data was lower than in Saxony. The hunting management unit with the highest wild boar density (1.5 wild boar/km^2^) was located in the west of the country (Figure 6). In the majority (393) of the 447 hunting management units, the number of wild boar /km^2^ did not exceed 1.0 wild boar/km^2^ (Figure 6).

## 4. Discussion

In the present study, the first 18 months of the respective ASF epidemics in Saxony and Latvia were analyzed and compared. The aim of the study was to describe the epidemiological situation of ASF in Saxony and to analyze the currently recommended control strategies. To amplify any possible effect, the data was compared with data analyses from the first 18 months of the ASF epidemic in a country that already had been affected before the currently available set of surveillance and control measures had been developed in detail. Latvia was chosen for comparison because ASF had presumably been introduced through migrating wild boar from an affected neighboring country, which resembled the situation in Saxony. Thus, both areas suffered from constant infection pressure due to migrating wild boar, some of which may have been infected with ASFV [4,10]. In addition, the availability of comprehensive surveillance data and good knowledge of the epidemiological situation made the comparison of these two study areas possible.

Despite the considerably smaller size of the study area in Saxony compared to Latvia, the number of samples investigated in the first 18 months after ASFV introduction was in Saxony more than twice as large as in Latvia. This could be due to the larger wild boar population density in Saxony, but it has been influenced also by different control strategies implemented in the two areas. Latvia was one of the first countries in the EU that was hit by the new ASF epidemic in 2014 [2,4,16]. Before ASF, genotype II had entered the first EU Member States and even during the first period of the epidemic, a common EU strategy for disease control in wild boar had not yet been elaborated. Thus, not only in Latvia but also in Lithuania and Estonia, which were also affected in 2014, most probably by the introduction by infected wild boar migrating across the border of affected neighboring countries, the disease spread through the whole country within two to three years [16]. In Poland, ASF was introduced in 2014 and is now present in wild boar populations in several regions, including a large area that has a common border with Saxony in Germany, without an indication that the epidemic might fade out [17]. In contrast, Saxony could benefit from the experience that had already been gained in other countries and had helped to eliminate ASF from affected wild boar populations in the Czech Republic and in Belgium [13,14,18].

In Saxony, a huge number of samples retrieved through passive surveillance originated from wild boar killed through an RTA; whereas, in Latvia, only six samples were obtained from wild boar that had been killed in an RTA. Yet, Schulz et al. [19] attributed the small number of samples from wild boar killed in an RTA rather to a reporting bias than to the actual occurrence of ‘road kills’. Due to the potential reporting bias, all wild boar sampled through passive surveillance (found dead, shot sick or involved in an RTA) were merged together and analyzed as originating from passive surveillance without including the exact cause of death of the wild boar. This approach may have biased the results. In a wild boar found dead, the probability of detecting ASFV is usually higher than in animals killed through an RTA [19,20,21]. Thus far, we are not aware of any conclusive evidence that ASF-infected animals have a higher chance to be killed in an RTA than healthy animals. Thus, merging data from wild boar found dead and killed in RTA may have led to underestimating the true ASFV prevalence in wild boar found dead. This could have been one of the reasons for the significantly lower ASFV prevalence in wild boar sampled in the course of passive surveillance in Saxony as compared to the ASFV prevalence in wild boar found dead in Latvia. In Saxony, almost the same numbers of samples originated from wild boar killed through an RTA than from wild boar found dead. Another reason for the lower ASFV prevalence in dead wild boar in Saxony could have been the active carcasses search that resulted in the detection of a larger number of dead wild boar compared to Latvia. In Latvia, active search for wild boar carcasses was originally not carried out systematically, since this measure had not yet been included in the control strategy at the time of disease introduction into Latvia. Furthermore, the period of time, during which the virus remained infectious in the carcasses and the contaminated environment, might have influenced the ASFV prevalence in the two areas. The temperature, soil composition and other factors affect the tenacity of the virus, thus the transmission rate and the prevalence [6,22]. The postmortem interval, i.e., the time between the death and the detection of these carcasses can influence the probability of transmission and thus the course of the epidemic [23].

The largest number of samples from both, active and passive surveillance was investigated in both countries 13–15 months after the introduction of ASF. Possibly, as the epidemic progressed, it became evident that the disease will not just disappear and more resources supporting the hunting effort and carcass searches were mobilized. Due to an increasing ASFV prevalence over time and the associated increase in mortality [24], it can be assumed that the number of dead wild boar generally increased, consequently resulting in a larger sample size. The increasing size of the affected area and the resulting larger number of ASFV-positive samples could also be seen in Poland [25]. The large sample size in November could also be observed in other countries [16] and is likely to be caused by the hunting season (driven hunts), which spans the winter months. A large number of samples from wild boar found dead in the summer months and also in November was comparable to the trend in the other two Baltic countries Estonia and Lithuania [16]. It is not surprising that the majority of samples were taken in the affected areas. Although the wild boar population density was found to be higher in other areas more centrally and westerly located, the hunting effort and the active carcasses search focused on the areas, where ASF had been newly introduced. EU legislation (Commission Implementing Regulation (EC) 2021/605) demands that all hunted wild boar and all detected carcasses need to be sampled in ASF-restricted areas, which obviously led to an increase in the sample size in affected areas.

The size of the affected area after the first 18 months of the ASF epidemic was clearly larger in Latvia than in Saxony. Due to the higher ASFV prevalence in Latvia, disease control was more difficult and thus, the virus had the chance to spread faster. One cornerstone of ASF control in Saxony was the erection of fences that were built up quickly and over long distances. In contrast, fences were not used in Latvia to control the spread of ASF in the wild boar population, since this measure had not yet been developed at that time. Saxony, therefore, had the advantage that the authorities could rely on the experience of other countries in controlling ASF, e.g., by fencing off affected areas and by implementing systematic wild boar carcass searches and disposing of the carcasses safely, thus reducing the amount of infectious ASFV present in the environment. At the time, when the first ASFV-positive wild boar was detected in Saxony, Belgium and the Czech Republic had already shown that intensive carcass searches supported by high financial rewards and fencing could reduce the spread of ASF and support disease elimination [13,14]. Thus, in contrast to Latvia, Saxony had the chance to prepare and to act immediately using measures that had been effective elsewhere, when ASF hit the region. It needs to be pointed out, however, that the number of affected wild boar is still growing and the affected areas increased in recent months. It seems, therefore, necessary to warrant that the required resources (personnel, finances, materials) remain continuously available until ASF is eliminated. This requires a high level of commitment from the State Government of Saxony, veterinary authorities, hunters and many others, as well as the critical evaluation of the measures and flexibility.

The rather low ASFV prevalence estimates in hunted wild boar and the high prevalence estimates in wild boar found dead in both study areas were not surprising and comparable to prevalence estimates in other countries [17,26,27]. The significantly higher ASFV prevalence estimates and the faster virus spread in Latvia probably supported the large number of outbreaks in domestic pigs in 2014 (32 outbreaks) and 2015 (10 outbreaks) (Animal Disease Information System, ADIS, of the European Union; accessed on 25 October 2022). Most of the affected farms in Latvia were small and poor biosafety/hygiene was identified as the most probable reason for virus introduction [4]. That could also be observed in Lithuania [28]. In Saxony, these kinds of pig holdings may be less frequent, which may have helped to prevent ASF outbreaks in domestic pig holdings in this region so far.

In Saxony, ASF seems to spread more slowly and despite the larger wild boar population density, a smaller area than in Latvia was affected by the disease after 18 months. Yet, ASF-positive wild boar carcasses are still regularly detected, indicating the constant need to further reduce the wild boar population in the areas at risk (areas geographically adjacent to an area with ASFV circulating in wild boar) and thus the number of susceptible hosts [29]. Further research is necessary to understand transmission dynamics and the maintenance of the virus within and between wild boar sub-populations, particularly if the wild boar population density is low [30].

Moreover, after one year, in which the virus only circulated in the eastern districts adjacent to the region affected in Poland, new cases emerged in the district of Meißen, approximately 65 km west of the initial cluster. This “jump” of ASF illustrates the continuous threat of a human-mediated disease spread. Even if the vast majority of people follow the rules meant to prevent ASF spread, ignorance or risky behavior of a single person can spark a new epidemic far away from previously ASF-affected regions and the best control measures cannot prevent such events. Therefore, to minimize these risks, it is inevitable to stay in contact with different groups of people, to actively distribute information and to raise awareness.

The course of the disease in Latvia, but also in Estonia, where it was thought for 1.5 years that the virus had been eliminated from the wild boar population [30,31], shows that ASF control in wild boar is very difficult. Thus far, no country in a comparable epidemiological situation has managed to successfully eliminate ASF from their country [10]. Although the epidemiological situation in Saxony cannot be compared to that in the Czech Republic or Belgium due to the constant infection pressure from western Poland, the comparison with Latvia has shown that the recently developed control strategies work, at least in principle, and have the potential to slow down the further spread of the disease. Accordingly, hope should not be given up that ASF can be successfully controlled through joint efforts and good national and international cooperation.

## Figures and Tables

**Table 1:** Composition of the analyzed ASF wild boar surveillance data from Saxony and Latvia for the first 18 months of ASF occurrence, as entered into the CSF/ASF wild boar surveillance database of the European Union (https://surv-wildboar.eu) (accessed on 11 July 2022).

		Saxony	Latvia
Study period		1 November 2020–27 April 2022	25 June 2014–30 November 2015
Number of analyzed data records		38,078	16,271
Size of the whole study area in km^2^		18,416	64,589
Number of samples from active surveillance		34,304	15,134
Number of samples from passive surveillance	Not specified	617	0
	Found dead	1611	1130
	Shot sick	147	1
	RTA	1399	6
Age of the sampled wild boar	Not specified	35,469	1265
	<1 year	1183	5615
	1–2 years	1062	9389
	>2 years	364	2

## Data Availability

The original data used for the analyses can be obtained from the corresponding author after approval by the responsible institutions in, Latvia and Saxony.

## Supplementary Materials

The following supporting information can be downloaded from: https://www.mdpi.com/article/10.3390/pathogens12010087/s1, Figure S1: ASFV prevalence estimates in hunted wild boar from Saxony and Latvia. The horizontal lines that form the top of the boxes illustrate the 75th percentile. The horizontal lines that form the bottom of the boxes represent the 25th percentile. The horizontal lines that intersect the box are the estimated median ASFV prevalences in hunted wild boar. Whiskers represent maximum and minimum values that are no more than 1.5 times the span of the interquartile range. Open circles represent outliers, which are single values greater or less than the extremes indicated by the whiskers.; Figure S2: ASFV prevalence estimates in wild boar found dead from Saxony and Latvia. The horizontal lines that form the top of the boxes illustrate the 75th percentile. The horizontal lines that form the bottom of the boxes represent the 25th percentile. The horizontal lines that intersect the box are the estimated median ASFV prevalences in wild boar found dead. Whiskers represent maximum and minimum values that are no more than 1.5 times the span of the interquartile range.
