# Supplementary material for: The First Eighteen Months of African Swine Fever in Wild Boar in Saxony, Germany and Latvia—A Comparison
Source: Pathogens. 2023 Jan 5;12(1):87. doi: 10.3390/pathogens12010087 (PMC9867452; doi:10.3390/pathogens12010087)
Supplement: Supplementary file 1 [file pathogens-12-00087-s001.zip › pathogens-2074550-supplementary.pdf]

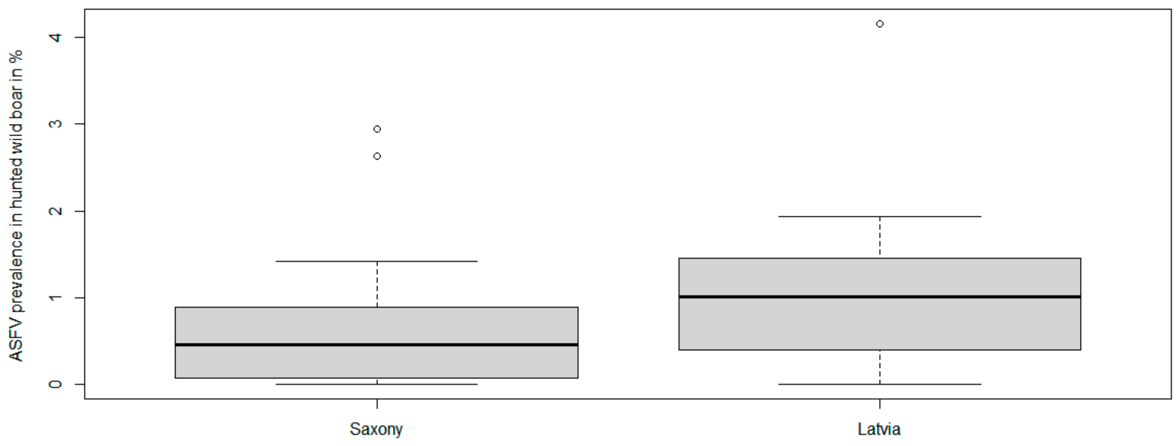

Figure S1: ASFV prevalence estimates in hunted wild boar from Saxony and Latvia. The horizontal lines that form the top of the boxes illustrate the 75th percentile. The horizontal lines that form the bottom of the boxes represent the 25th percentile. The horizontal lines that intersect the box are the estimated median ASFV prevalences in hunted wild boar. Whiskers represent maximum and minimum values that are no more than 1.5 times the span of the interquartile range. Open circles represent outliers, which are single values greater or less than the extremes indicated by the whiskers.

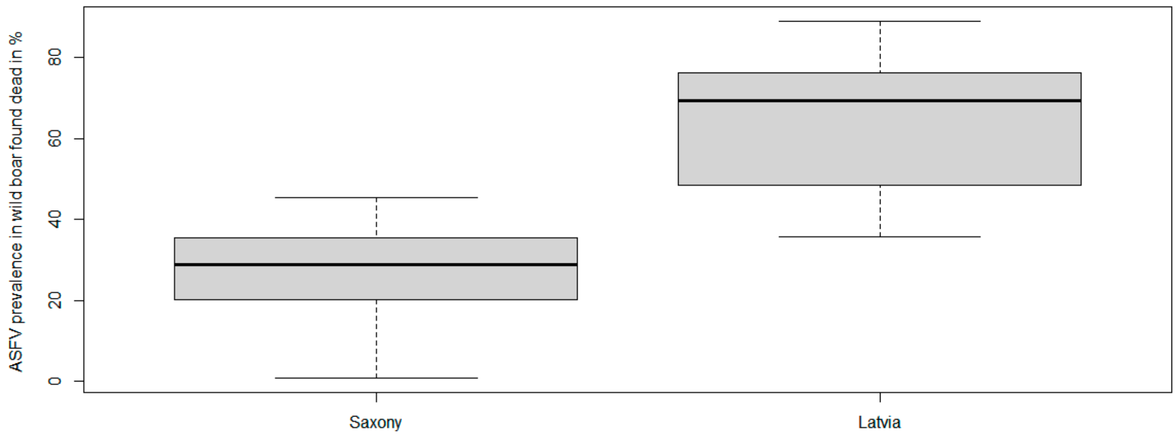

Figure S2: ASFV prevalence estimates in wild boar found dead from Saxony and Latvia. The horizontal lines that form the top of the boxes illustrate the 75th percentile. The horizontal lines that form the bottom of the boxes represent the 25th percentile. The horizontal lines that intersect the box are the estimated median ASFV prevalences in wild boar found dead. Whiskers represent maximum and minimum values that are no more than 1.5 times the span of the interquartile range.
